# Cupping and Ice in Acute Gastritis

**Published:** 1876-11

**Authors:** S. D. Chase

**Affiliations:** Osage, Iowa


### CUPPING AND ICE IN ACUTE GASTRITIS.

By S. D. CHASE, M. D., of Osage, Iowa.

Returning home Monday evening, March 27, 1876, badly chilled from an extensive ride into the country, I ate a hearty supper, and went out to visit some patients in the city. Although I began to feel distress directly after eating, I was able to very nearly complete my calls before I was compelled to return, at 11 P. M. Being greatly fatigued, I retired, and immediately fell asleep, to awake in an hour with a terrible gastric pain in the splenic portion of the stomach.

In half an hour I began to vomit its sour and acrid contents. I took an alkali and some lactopeptine, which neutralized the acidity, but did not check the vomiting or relieve the pain. This continuing to increase, I applied sinapisms freely over the epigastrium, and began the use of morph. sulph., with small doses of submur. hyd., dry, on the tongue. The vomiting partially abated, but the pain continued, compelling the free use of anodynes.

Active counter-irritation was constantly maintained, and opium taken in large doses, with such a mitigation of the symptoms as gave a confident hope of relieving the congestion; yet, in defiance of the most energetic contra efforts, it passed rapidly into inflammation. The usual treatment was employed, and vigorous exertions were made to allay the early-developed heat. As the stomach had rejected everything, an unsuccessful attempt was made to alleviate by cold externally.

Thus the conflict raged until the next Monday, when the indications, to me, were that the struggle was most over, unless I could get immediate relief. The heat was so intense that small pieces of ice would be ejected with great force the instant swallowed, the water melted from them following in foam.

At this stage Dr. Whitley, under whose care I was, summoned Dr. Blackman, of West Mitchell, in consultation, who decided, as had Dr. Whitley, that the only hope was *to force ice into my stomach, and compel its retention.*

Dr. Blackman then prepared large cups, so that, when ignited, a volume of flame streamed from them two to three feet.

Then, like Pyrrhus in the palace of Priam, with a whirl he brought them down upon my swollen stomach. For an instant it almost seemed as though soul and body would separate, so great was the soreness, both external and internal; yet each application gave relief. Although accustomed to using cups, and seeing them used by others, I never before realized their wondrous power.

After thus applying a number of them, they began to feed me with finely-shaven ice, literally gorging me, as our good grand dames do turkeys for Christmas fattening. The desire to vomit seemed irrisistible, yet to do so appeared impossible. The cupping and ice diet were continued an hour, changing the cups every ten to fifteen minutes, until my entire gastric region looked as though it had been the focus of a battering-ram.

At the close of the first half hour all inclination to vomit ceased. In three quarters my stomach appeared saturated, and the melted ice began to trickle through the pylorus. An almost incredible amount of ice was taken during the following five days and nights, my stomach craving and tolerating it. Near the close of the fifth day, I commenced to take water with the ice.

Saturday morning I was so greatly relieved that I thought the danger passed, when, without premonition, I sank suddenly into a cold sweat, which continued until Monday morning, very nearly taking what vitality remained. Although so grievous to endure, this had a "silver lining," as the heat and pain, which had continued "an abiding affliction," gradually passed away with the sweating.

Tuesday the mucous membrane began to slough in my mouth, and extending into my stomach appeared to slough deeply there, occasioning an intense, burning, stinging pain, which continued until Saturday. The ice had probably something to do with this. My mouth was terribly burned, my tongue deeply fissured, and the week a very distressing one. The following was more comfortable, but on Monday of the first week I was somewhat startled by strong indications of gastric ulceration and gangrene. This yielded to carbo ligni.

The last week was much like the preceding, my tongue giving me the most discomfort; but little heat and pain remained in my stomach, as I did not vex it with anything, ice water being all I attempted to take. On the thirty fifth day I made the first successful attempt to take nourishment, every previous endeavor having given distress, the stomach not even tolerating gum-acacia water, or that of ulmi cort.

A nourishing enema was given me the second week, but as it created nausea it was not repeated, my continuing strength rendering it unnecessary. During the entire five weeks I did not take as much nutriment as one-third of a cup of milk would furnish, and this to my detriment. On the thirty-fourth day I felt the first symptoms of exhaustion. I think my vital energy would have supported me another week, yet I was very glad not to be compelled to try it.

While I would have preferred to study the symptoms and treatment of this distressing and dangerous disease under other circumstances, yet to watch its progress and note its changes afforded me an interesting theme for reflection. After the second week I took little medicine. Circulation moderate most of the time, attributable doubtless to the nausea. Respiration rapid, necessitating a continued low temperature. I do not find this disease very satisfactorily treated in medical works. Dr. Flint's is the best.

I have now been "on my food" three weeks, and, contrary to my expectation, my stomach does duty well. I can, with caution, take toast and very nearly raw beef with satisfaction and impunity.

